# Integrated analysis of genes shared between type 2 diabetes mellitus and osteoporosis

**DOI:** 10.3389/fphar.2024.1388205

**Published:** 2024-06-20

**Authors:** Fangyu Li, Ying Wang, Jie Cao, Qi Chen, Yuanyuan Gao, Rui Li, Li Yuan

**Affiliations:** Department of Endocrinology, Union Hospital, Tongji Medical College, Huazhong University of Science and Technology, Wuhan, China

**Keywords:** type 2 diabetes mellitus, osteoporosis, biomarker, LASSO, VNN1

## Abstract

**Background:**

The relationship between type 2 diabetes mellitus (T2DM) and osteoporosis (OP) has been widely recognized in recent years, but the mechanism of interaction remains unknown. The aim of this study was to investigate the genetic features and signaling pathways that are shared between T2DM and OP.

**Methods:**

We analyzed the GSE76894 and GSE76895 datasets for T2DM and GSE56815 and GSE7429 for OP from the Gene Expression Omnibus (GEO) database to identify shared genes in T2DM and OP, and we constructed coexpression networks based on weighted gene coexpression network analysis (WGCNA). Shared genes were then further analyzed for functional pathway enrichment. We selected the best common biomarkers using the least absolute shrinkage and selection operator (LASSO) algorithm and validated the common biomarkers, followed by RT-PCR, immunofluorescence, Western blotting, and enzyme-linked immunosorbent assay (ELISA) to validate the expression of these hub genes in T2DM and OP mouse models and patients.

**Results:**

We found 8,506 and 2,030 DEGs in T2DM and OP, respectively. Four modules were identified as significant for T2DM and OP using WGCNA. A total of 19 genes overlapped with the strongest positive and negative modules of T2DM and OP. Kyoto Encyclopedia of Genes and Genomes (KEGG) analysis showed these genes may be involved in pantothenate and CoA biosynthesis and the glycosaminoglycan biosynthesis-chondroitin sulfate/dermatan sulfate and renin-angiotensin system signaling pathway. The LASSO algorithm calculates the six optimal common biomarkers. RT-PCR results show that *LTB*, *TPBG,* and *VNN1* were upregulated in T2DM and OP. Immunofluorescence and Western blot show that *VNN1* is upregulated in the pancreas and bones of T2DM model mice and osteoporosis model mice. Similarly, the level of *VNN1* in the sera of patients with T2DM, OP, and T2DM and OP was higher than that in the healthy group.

**Conclusion:**

Based on the WGCNA and LASSO algorithms, we identified genes and pathways that were shared between T2DM and OP. Both pantothenate and CoA biosynthesis and the glycosaminoglycan biosynthesis-chondroitin sulfate/dermatan sulfate and renin–angiotensin systems may be associated with the pathogenesis of T2DM and OP. Moreover, *VNN1* may be a potential diagnostic marker for patients with T2DM complicated by OP. This study provides a new perspective for the systematic study of possible mechanisms of combined OP and T2DM.

## 1 Introduction

Type 2 diabetes mellitus (T2DM) is the most common metabolic disease and is mainly characterized by metabolic disorders, such as hyperglycemia, hyperlipidemia, and insulin resistance. Although the main target organs damaged by long-term diabetes are the heart, brain, nerves, eyes, and kidneys, the musculoskeletal system has also received attention in recent years (Wen et al., 2021; Hofbauer et al., 2022; Sheu et al., 2022). Osteoporosis (OP) is characterized by low bone mass and microarchitectural deterioration of the bone tissue, leading to reduced bone strength and increased risk of low-energy fractures or fragility fractures (Anam and Insogna, 2021). The prevalence of T2DM and osteoporosis due to an aging population is rapidly increasing and will soon become a global epidemic, imposing an overwhelming burden on healthcare systems (Lane, 2006; Yamamoto et al., 2009; Napoli et al., 2017; Carey et al., 2022; Sun et al., 2022).

Increasingly, studies have proposed that T2DM is associated with poor bone health, resulting in an increased risk of fractures and worse post-fracture outcomes (Madsen et al., 2019; Anagnostis et al., 2020; Di Monaco et al., 2023). The pathophysiologic mechanisms underlying bone fragility in diabetic patients are complex and poorly understood. Hyperglycemia, oxidative stress, and accumulation of advanced glycation end products, inflammatory cytokines, myogenic hormones, and incretins, hydrogen sulfide (H_2_S) production and cortisol secretion, peripheral activation, and sensitization may all alter bone formation and bone resorption, impair collagen properties, and increase bone marrow fat, ultimately leading to decreased bone strength (Zhukouskaya et al., 2015; Napoli et al., 2017; Eller-Vainicher et al., 2019; Eller-Vainicher et al., 2020). In addition, the typical complications of T2DM (retinopathy, nephropathy, and cardiovascular disease) and the increased likelihood of falls may partly account for the increased risk of fractures (Napoli et al., 2017).

More importantly, previous meta-analyses have identified some genetic overlap between T2DM and OP. For example, KLHDC5 and CDKAL1 are not only associated with femoral neck bone mineral density (BMD) (FN_BMD) (*p*-value of KLHDC5 = 1.90E−12, *p*-value of CDKAL1 = 2.70E−13) (Estrada et al., 2012) but also with T2DM (*p*-value of KLHDC5 = 6.1E−10, *p*-value of CDKAL1 = 6.00E−36) (Voight et al., 2010; Morris et al., 2012). Although these studies have identified potential multiple effector genes between T2DM and osteoporosis, there has been no systematic search for shared genes affecting these two diseases. Moreover, the etiology and pathogenesis of these two diseases are very complex, and there are no sufficiently robust programs to prevent or treat both diseases. Therefore, finding a link between T2DM and OP may provide new ideas for the diagnosis and treatment of both diseases.

To elucidate common biomarkers and enriched pathways in T2DM and OP mechanisms, we employed multiple integrative bioinformatics tools to reveal the hub genes and potential mechanism underlying diabetic osteoporosis by collecting two T2DM datasets and two OP datasets from the Gene Expression Omnibus (GEO) database. A diagnostic model for predicting diabetic osteoporosis was constructed based on the least absolute shrinkage and selection operator (LASSO) logistic regression algorithm of the hub genes identified in the T2DM- and OP-related pathogenic genes. We took the intersection of bioinformatics and machine-learning hub genes to obtain shared hub genes. Finally, we validated the expression of the co-hub genes using T2DM and OP animal models.

## 2 Materials and methods

### 2.1 Data selection

We obtained the GSE76894 and GSE76895 through the NCBI GEO public database (http://www.ncbi.nlm.nih.gov/geo/). Data for 19 T2DM patients and 84 control samples from GSE76894, 36 T2DM patients, and 32 control samples from GSE76895. Two microarray data were based on the GPL570 (HG-U133_Plus_2) Affymetrix Human Genome U133 Plus 2.0 Array (Affymetrix, Santa Clara, CA, United States).

Two OP-related datasets, GSE56815 and GSE7429, were also selected from the GEO website, which was done in the same way. The samples in the GSE56815 and GSE7429 datasets are osteoporosis patients characterized by low BMD. Dataset GSE56815 includes 40 female controls (20 premenopausal, 20 postmenopausal) and 40 female patients with low BMD (20 premenopausal, 20 postmenopausal). Dataset GSE7429 includes 10 postmenopausal controls (age 56.8 ± 1.93 years) and 10 postmenopausal patients (age 57.9 ± 1.45 years) with low BMD. Microarray data were based on the GPL96 [HG-U133A] Affymetrix Human Genome U133A Array (Affymetrix, Santa Clara, CA, United States).

### 2.2 Data processing

We combined two datasets for each disease to increase the sample size. R software (version 4.2.3; https://www.r-project.org/) and used the “limma” package in the R software to screen for DEGs between the control and T2DM groups and between the control and OP groups, respectively, with parameters set to adj. *p*-value <0.05. We then used the “ggplot2” and “heat map” packages to draw volcano maps and heat maps. DEGs with log2FC > 0 were considered to be upregulated genes, while the downregulated genes were screened according to log2FC < 0.

### 2.3 Weighted gene coexpression network analysis (WGCNA)

WGCNA, a method for identifying highly co-expressed gene modules and their relationship with disease (Rasmussen et al., 2020), utilizes WGCNA software package R.4.2.3 for analysis. In this study, the WGCNA R package was used to construct coexpression networks integrating clinical characteristics for the DEGs of T2DM and OP. Initially, hierarchical cluster analysis using the Hclust function in R was conducted to eliminate outlier samples. Subsequently, an adjacency matrix was established using a soft-threshold β and a gene–gene correlation matrix to describe the degree of association between the nodes. The resultant clusters were analyzed via a topological overlap matrix, assigning modules by color and module feature (ME). Furthermore, Pearson’s correlation test was applied to calculate the correlation between ME and clinical features. The modules with a |ME|> 0.3 and a *p*-value <0.05 were deemed to have significant interactions with clinical features (Zhang et al., 2023).

### 2.4 Enrichment analysis

The potential shared genes were identified as T2DM-related DEGs overlapped with the OP-related ones. These shared genes could have the potential ability to link the pathogeneses of T2DM and OP.

To further determine the biological features of potentially shared genes, Gene Ontology (GO) analysis was accomplished by the Database for Annotation, Visualization and Integrated Discovery (DAVID) online tool. The mission of the DAVID knowledgebase is to support biomedical and other research by providing comprehensive information about the evolution of protein-coding gene families, particularly protein phylogeny, function, and genetic variations impacting that function. GO enrichment analysis was performed using the DAVID online tool, including molecular functions (MFs), cellular components (CCs), and biological processes (BPs). The Encyclopedia of Genes and Genomes (KEGG) was used for pathway enrichment analyses (https://bioinfo.org/kobas/). The enriched function with *p* < 0.05 was considered a significant pathway. Based on this analysis method, we selected the top 15 GO biological processes and 10 KEGG pathways.

### 2.5 Identification of optimal diagnostic shared genes

To better screen the risk of crosstalk genes between T2DM and OP, LASSO regression was performed in R project. The Co-DEGs of T2DM and OP were retained for feature selection, and the best tandem genes were initially identified using LASSO regression. To further narrow the scope, we combined the two results of this LASSO algorithm by taking the intersection set.

### 2.6 T2DM and OP mouse model

Six-week-old male C57BL/6J WT mice were randomly divided into two groups (n = 5 or 6 mice/group): (1) standard diet control group (SD) and (2) HFD + streptozotocin-treated group (HFD + STZ). Mice in the SD group were fed a standard chow diet. In the HFD + STZ group, mice were first fed an HFD (#D12492) for 4 weeks and then injected with STZ (30 mg/kg, Sigma-Aldrich) for three consecutive days to induce T2DM, as previously described (Ma et al., 2020). The mice with blood glucose levels over 16.7 mmol/L measured by a blood glucose meter (LifeScan) were considered to have T2DM and were continually an HFD until 16 weeks.

Eight-week-old female C57BL/6J WT mice were randomly divided into two groups (n = 5 or 6 mice/group): 1) sham surgery and 2) ovariectomy (OVX). The mice were anesthetized by intraperitoneal injection with 1% sodium pentobarbital solution (50 mg/kg), and the bilateral ovaries were removed through a dorsal approach (Shen et al., 2020). After finding the ovary, the ovaries of the mice in the sham group were returned to the abdominal cavity without resection.

All mice were housed in a specific pathogen-free (SPF) animal laboratory in a 12-h light:12-h dark cycle at room temperature (20°C–22°C). All experiments were performed according to procedures approved by the Animal Research Committee of Tongji Medical College, Huazhong University of Science and Technology, Hubei Province, China (Reference Number: 3,053, Date Approved: 10 October 2020).

### 2.7 Blood glucose levels, intraperitoneal insulin tolerance tests, and intraperitoneal glucose tolerance tests

Tail fasting blood glucose was assessed in mice using a blood glucose meter (LifeScan) following a 12-hour fast. For the intraperitoneal glucose tolerance test (IPGTT), mice underwent injection with glucose (2 g/kg body weight, i.p.) after a 12-hour fast, with blood samples collected from the tail vein at 0 min, 30 min, 60 min, 90 min, and 120 min post-injection. Similarly, the intraperitoneal insulin tolerance test (IPITT) involved injecting insulin (0.75 U/kg body weight, i.p.) into fasted mice, with blood samples collected from the tail vein at 0 min, 15 min, 30 min, 60 min, and 90 min thereafter.

### 2.8 Immunofluorescence staining

Paraffin tissue paraffin sections were subjected to deparaffinization with xylene, followed by dehydration in a series of 100%, 95%, 85%, and 70% ethanol. The sections were incubated overnight at 4°C with antibodies specific for anti-insulin (1:100, Abcam, U.K.), glucagon (1:100, Abcam, U.K.), vanin1 (1:100, Proteintech, China), and osteopontin (1:100, R&D Systems, United States). After three washes with PBS for 10 min, the sections were incubated with fluorescent secondary antibody (1:100 dilution) for 1 h, and nuclei were counterstained with DAPI (Servicebio, Wuhan, China) at room temperature for 10 min in the dark (Chen et al., 2023a). The sections were observed by a fully automated slice scanning system (VS120, Olympus, Japan).

### 2.9 Western blotting

Total protein in cell lysates was extracted using a radio-immunoprecipitation assay (RIPA) buffer containing protease and phosphatase inhibitors. Tissue lysates were boiled in SDS loading buffer for 10 min; proteins were then resolved by SDS-PAGE and transferred to polyvinylidene difluoride membranes (PVDF, Millipore Corp, MA, United States). After incubation with 5% BSA in TBST for 1 h, the membranes were incubated with anti-VNN1 antibody (1:1000, Proteintech, China) at 4 °C overnight. The membranes were washed three times with TBST and then incubated with horseradish peroxidase-conjugated anti-rabbit antibody (1:2000 dilution) for 1 h.

### 2.10 Micro-CT analysis

The three-dimensional structure of trabecular bone was analyzed in the femur on a micro-CT scanner (Skyscan-1176, Bruker, Karlsruhe, Germany). The system was configured with a 1-mm AL filter, 9 μm resolution, 50 kV voltage, and 200 μA current. For the femur, scanning started at the lower growth plate and extended proximally for 600 slices, which included trabecular bone (slices 1–400) and cortical bone (slices 500–550). The following parameters were measured by CT analyzer (version 1.15.4.0): bone volume (BV), total volume (TV), trabecular bone volume fraction (BV/TV), trabecular bone thickness (Tb.Th), trabecular bone number (Tb.N), trabecular bone separation (Tb.Sp), structural model index (SMI), and cortical bone thickness (Cb.Th).

### 2.11 RT-PCR

Total RNA was extracted from mouse pancreas and bone tissues using TRIzol reagent, and cDNA was reverse-transcribed using the HiScript III RT SuperMix (Vazyme, China) under the following conditions: 15 min at 37 °C and 5 s at 85 °C. Next, the cDNA was amplified using ChamQ SYBR qPCR Master Mix (Vazyme, China). β-Actin was used as an internal control in 10 μL volume. The 2^−ΔΔCq^ method was used to calculate the relative mRNA levels. The relative mRNA levels were calculated by the 2^−ΔΔCq^ method, and the primer sequences are shown in Table 1.

**TABLE 1:** mRNA primer sequence.

Gene name	Primer sequence (5′–3′)
*LGALS2-*F	AAC​ATG​AAA​CCA​GGG​ATG​TCC
*LGALS2*-R	CGA​GGG​TTA​AAA​TGC​AGG​TTG​AG
*LTB*-F	TGG​CAG​GAG​CTA​CTT​CCC​T
*LTB*-R	TCC​AGT​CTT​TTC​TGA​GCC​TGT
*STEAP4*-F	GGG​AAG​TCA​CTG​GGA​TTG​AAA​A
*STEAP4*-R	CCG​AAT​AGC​TCA​GGA​CCT​CTG
*TPBG*-F	CGG​CAA​CCA​CCT​GAA​GGA​A
*TPBG*-R	AAG​GCC​ACC​ATA​CCC​TCG​AA
*VGF*-F	TTC​AGT​CCG​AGC​AAT​GCT​AAG
*VGF*-R	AGC​CTG​GAA​TTG​GGA​AGG​GA
*VNN1*-F	CTT​TCC​TCG​CGG​CTG​TTT​AC
*VNN1*-R	CCT​CCA​GGT​ATG​GGT​AGA​TCG​T

### 2.12 Enzyme-linked immunosorbent assay (ELISA)

The VNN1 levels were determined using the Human Mouse Vanin 1 (VNN1) ELISA Kit (Jianglai, China) according to the manufacturer’s instructions. Absorbance at 450 nm was detected using an enzyme labeler (PerkinElmer, Waltham, MA, United States).

### 2.13 Clinical patient selection

The clinical samples involved in the experiment were all from our previous studies (Chen et al., 2023b). The study was conducted according to the guidelines of the Declaration of Helsinki and approved by the Ethics Committee of Tongji Medical College, Huazhong University of Science and Technology (ChiCTR2000034751). All subjects gave their informed consent for inclusion before they participated in the study.

Type 2 diabetes mellitus group: 1) typical diabetes mellitus symptoms (irritable thirst and excessive drinking, polyuria, polyphagia, and unexplained weight loss); 2) random blood glucose ≥11.1 mmol/L; 3) fasting blood glucose ≥7.0 mmol/L; 4) blood glucose after glucose loading for 2 h ≥ 11.1 mmol/L; and 5) glycated hemoglobin ≥6.5%, with two of the above conditions fulfilled, and exclusion of other types of diabetes.

Based on the diagnostic criteria of the International Society for Clinical Densitometry (Watts et al., 2013), we measured BMD in participants by dual-energy X-ray absorptiometry (DXA) and calculated T-scores. We then categorized participants into normal (T-score ≥ −1), osteoporotic (−2.5 < T-score < −1), and osteopenia (T-score ≤ −2.5) according to T-scores. In this study, osteoporosis was diagnosed as femoral neck BMD ≤ −2.5 SD measured by DXA.

Type 2 diabetes mellitus combined with osteoporosis (T2DM + OP) group: 1) meet the diagnosis of type 2 diabetes mellitus; 2) meet the diagnosis of osteoporosis.

### 2.14 Statistical analysis

The data were analyzed using the GraphPad Prism software (8.0.1). Differences in numerical parameters between the two groups were assessed by an unpaired two-tailed t-test. All data are expressed as the mean ± SD, and *p* < 0.05 was considered statistically significant.

## 3 Results

### 3.1 DEGs identification

**FIGURE 1:**
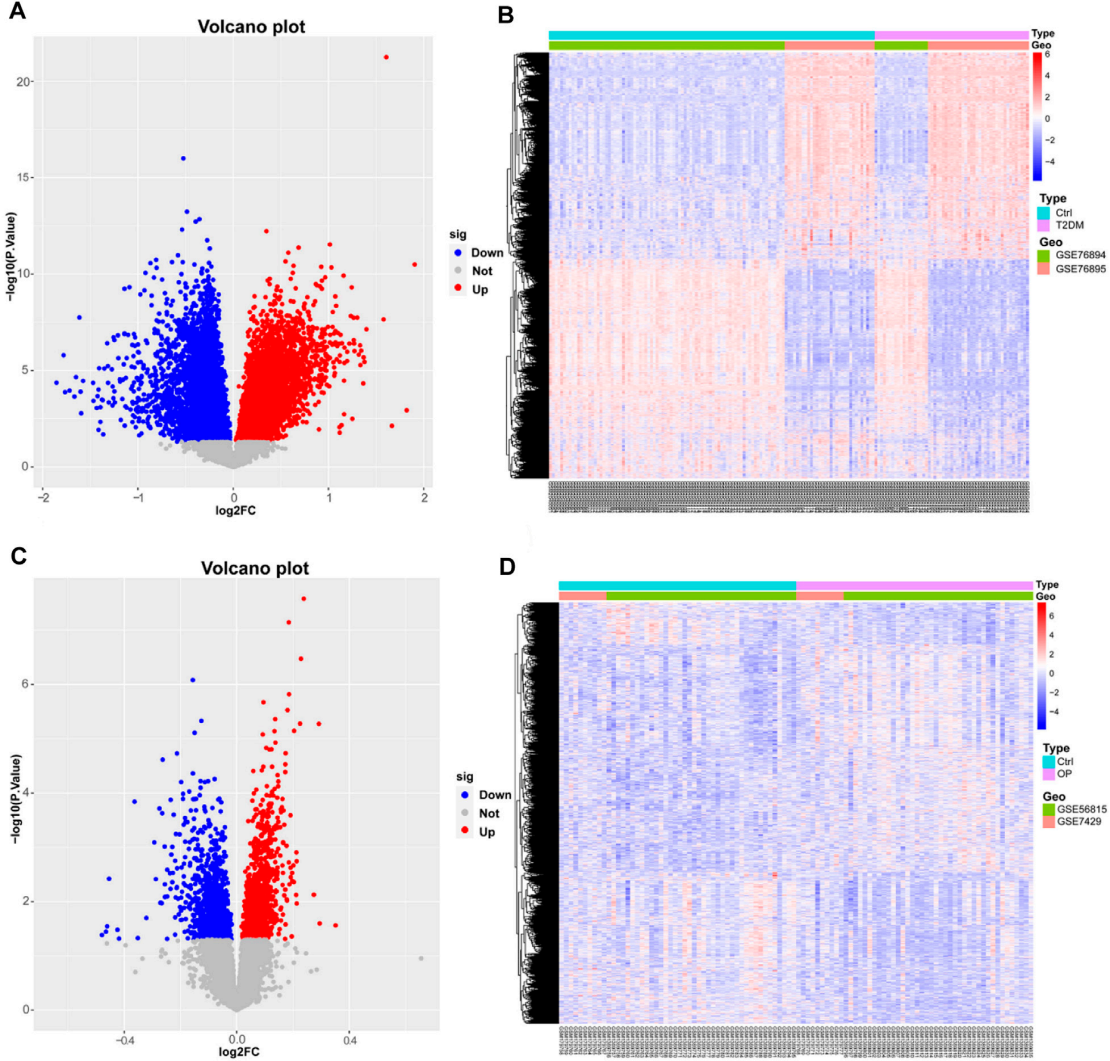
Identification of differentially expressed genes in T2DM and OP. **(A and B)** Volcano map and heat map of DEGs for T2DM. **(C and D)** Volcano map and heat map of DEGs for OP. DEGs, differentially expressed genes; T2DM, type 2 diabetes mellitus; OP, osteoporosis.

Two microarray datasets (GSE76894 and GSE76895) of T2DM were collected from the GEO database. The datasets comprised data from 19 T2DM patients and 84 control samples from GSE76894 and 36 T2DM patients and 32 control samples from GSE76895. To identify DEGs between T2DM and healthy controls, we gathered microarray expression profiles of GSE76894 and GSE76895 from the GEO database. Following the consolidation and normalization of the microarray data, we identified 8,506 DEGs between T2DM and healthy controls utilizing the “limma” package (*p* < 0.05), comprising 4,121 upregulated genes and 4,385 downregulated genes. Subsequently, we generated a volcano map (Figure 1A) and a hierarchically clustered heat map of the DEGs (Figure 1B).

We also selected two OP datasets (GSE56815 and GSE7429) from the GEO database and processed them with the same method, which resulted in 2,030 DEGs, including 1,091 upregulated genes and 939 downregulated genes. The results were plotted as a volcano map (Figure 1C) and a heat map (Figure 1D).

### 3.2 Construction and module analysis of weighted gene coexpression network (WGCNA)

**FIGURE 2:**
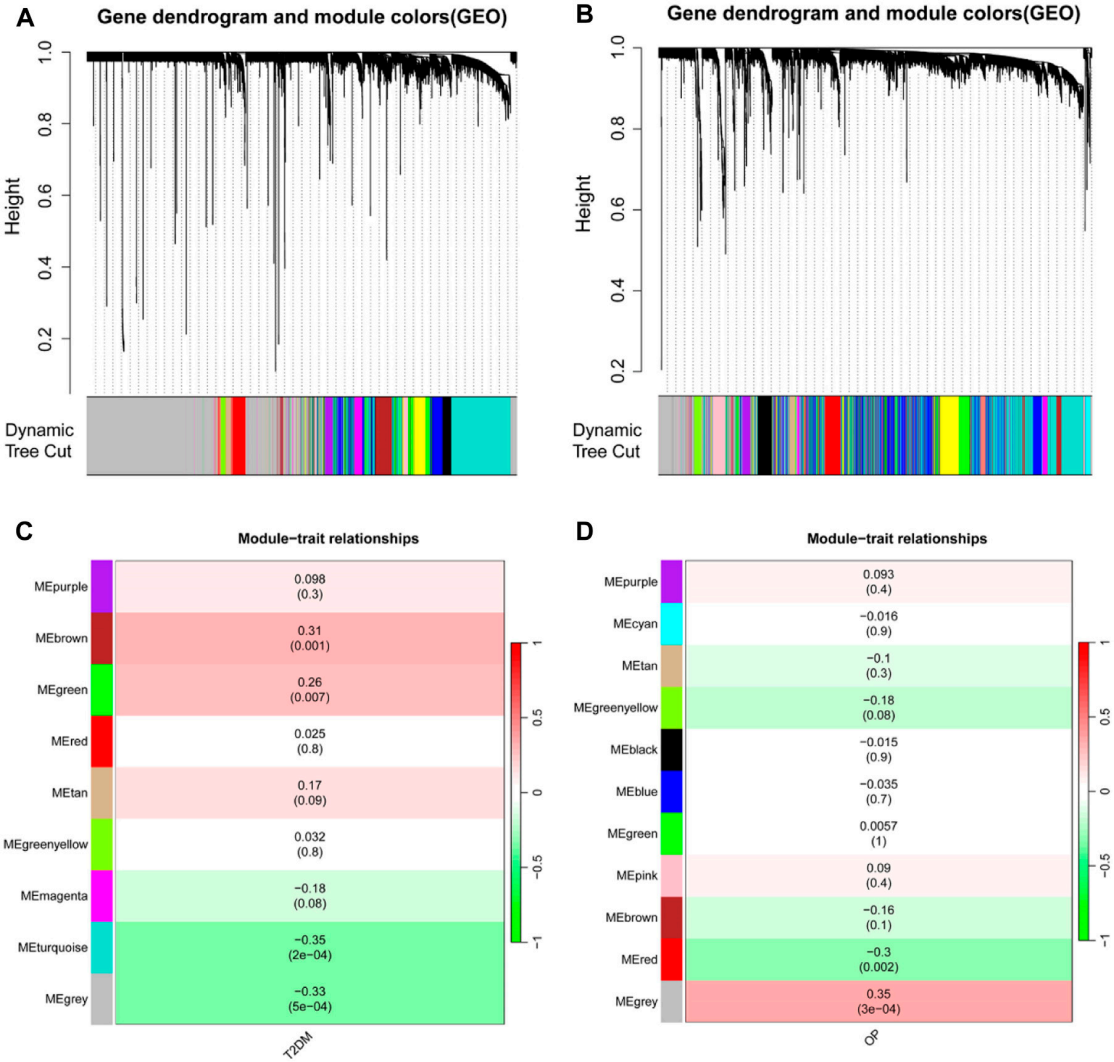
Identification of modules linked to clinical features of T2DM and OP by WGCNA. **(A and B)** Cluster dendrogram of co-expressed genes in T2DM **(A)** and OP **(B)**. **(C and D)** Heap of module–trait relationships in T2DM **(C)** and OP **(D)**. T2DM, type 2 diabetes mellitus; OP, osteoporosis; WGCNA, weighted gene coexpression network analysis.

A WGCNA was used to detect clusters of co-expressed genes exhibiting expression differences between T2DM and OP and to assess the correlation of combined modules with disease phenotypes. The T2DM model had a soft-threshold β = 16, whereas the OP model had a soft-threshold β = 8 (Figures 2A, B). After combining similar gene modules, nine modules were recognized in the T2DM model set and 11 modules in the OP model set. As shown in Figure 2C, the brown module had the highest positive correlation with the occurrence of T2DM (r = 0.31), and the turquoise module had a substantial negative correlation with the occurrence of T2DM (r = −0.35). Furthermore, in the OP modeling set, the gray module demonstrated the strongest positive correlation with OP occurrence (r = 0.35), and the red module showcased the most prominent negative correlation with OP occurrence (r = −0.3) (Figure 2D).

### 3.3 Identification of shared pathways

**FIGURE 3:**
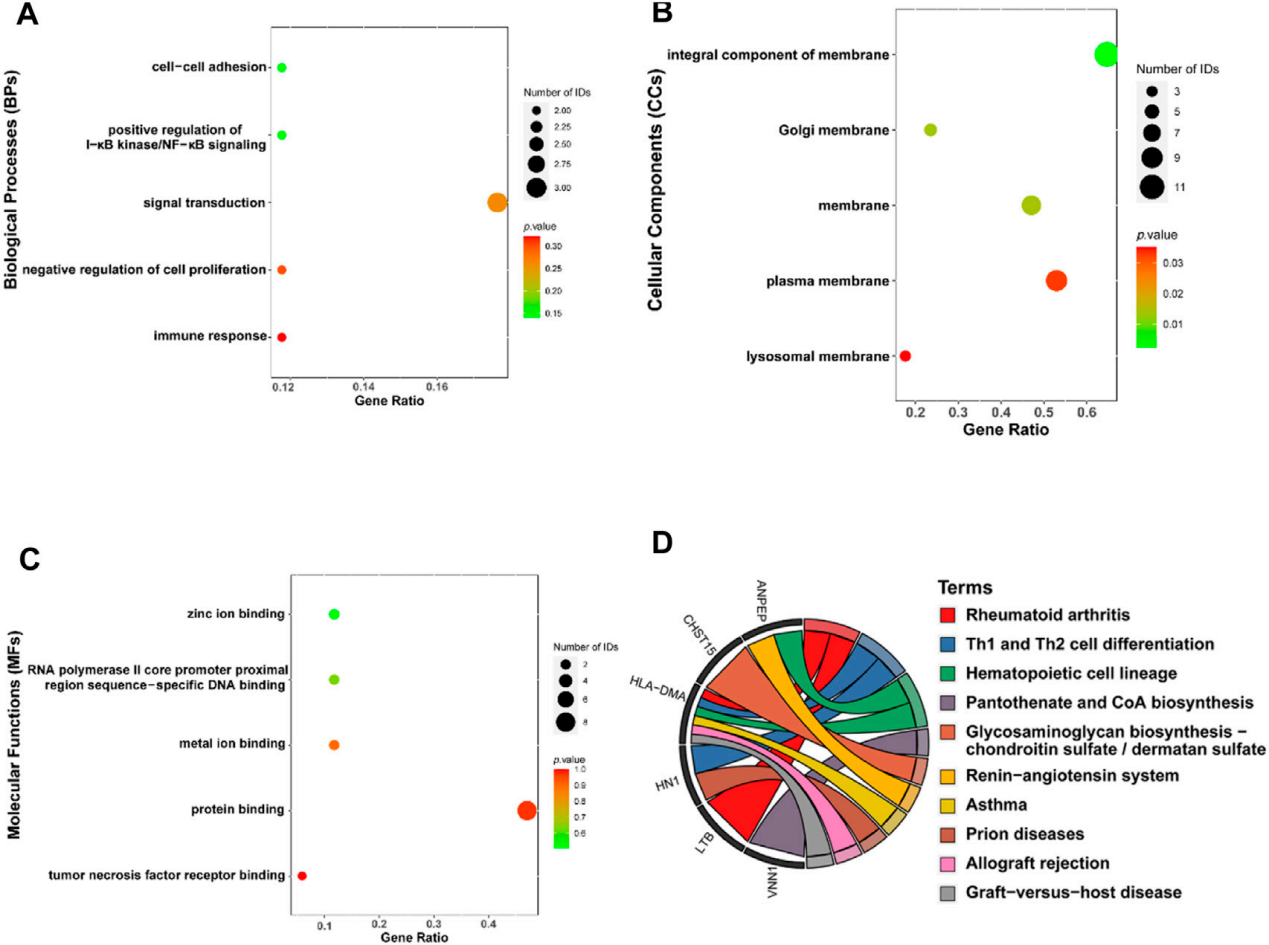
Functional enrichment analysis for the co-DEGs. Molecular functions **(A)**, cellular components **(B)**, biological processes **(C)**, and KEGG **(D)** of co-DEGs. Co-DEGs, common differentially expressed genes; KEGG, Kyoto Encyclopedia of Genes and Genomes.

A total of 19 genes overlapped in the strongest positive and negative modules of T2DM and OP. These genes may be related to the pathogenesis of T2DM and OP. We used the DAVID online tool to perform GO analysis of these 19 co-DEGs, including biological processes (BPs), cellular components (CCs), and molecular functions (MFs) (Figures 3A–C). The BPs were mainly focused on the cell–cell adhesion, positive regulation of I-κB kinase/NF-κB signaling, signal transduction, negative regulation of cell proliferation, and immune response; CCs were mainly focused on the integral component of the membrane and the Golgi, plasma, and lysosomal membranes; and MFs were mainly focused on zinc ion binding, RNA polymerase II core promoter proximal region sequence-specific DNA binding, metal ion binding, protein binding, and tumor necrosis factor receptor binding. We then performed KEGG enrichment analysis using the KOBAS website and identified 10 KEGG pathways (Figure 3D). The KEGG pathway analysis showed that the DEGs were closely related to pantothenate and CoA biosynthesis and the glycosaminoglycan biosynthesis–chondroitin sulfate/dermatan sulfate, renin–angiotensin system, and other pathways.

### 3.4 Prediction of optimal diagnostic genes

**FIGURE 4:**
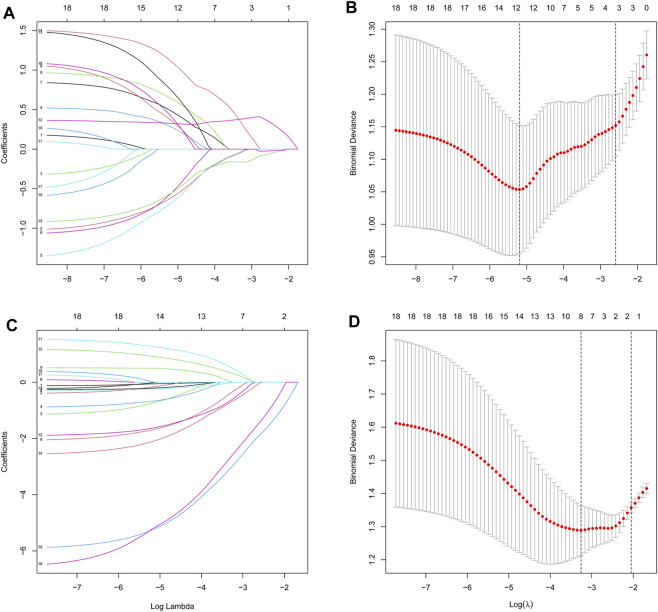
Identification of diagnostic feature biomarkers using the LASSO algorithm. Optimal diagnostic gene selection by LASSO regression for T2DM **(A and B)** and OP **(C and D)**. T2DM, type 2 diabetes mellitus; OP, osteoporosis; LASSO, least absolute shrinkage and selection operator.

We then extracted the expression data of the 19 genes from the T2DM gene expression profile using the LASSO algorithms with an optimal lambda value of lambda.min = 0.0055 (Figures 4A, B). The results from the LASSO regression identified 12 genes as signature genes in T2DM. We used the same method to obtain eight genes for the diagnosis of OP (lambda.min = 0.0385; Figures 4C, D). The diagnostic genes of the two diseases were intersected to obtain six common diagnostic genes, namely, *LGALS2*, *LTB*, *STEAP4*, *TPBG*, *VGF*, and *VNN1*.

### 3.5 Validation of hub genes

**FIGURE 5:**
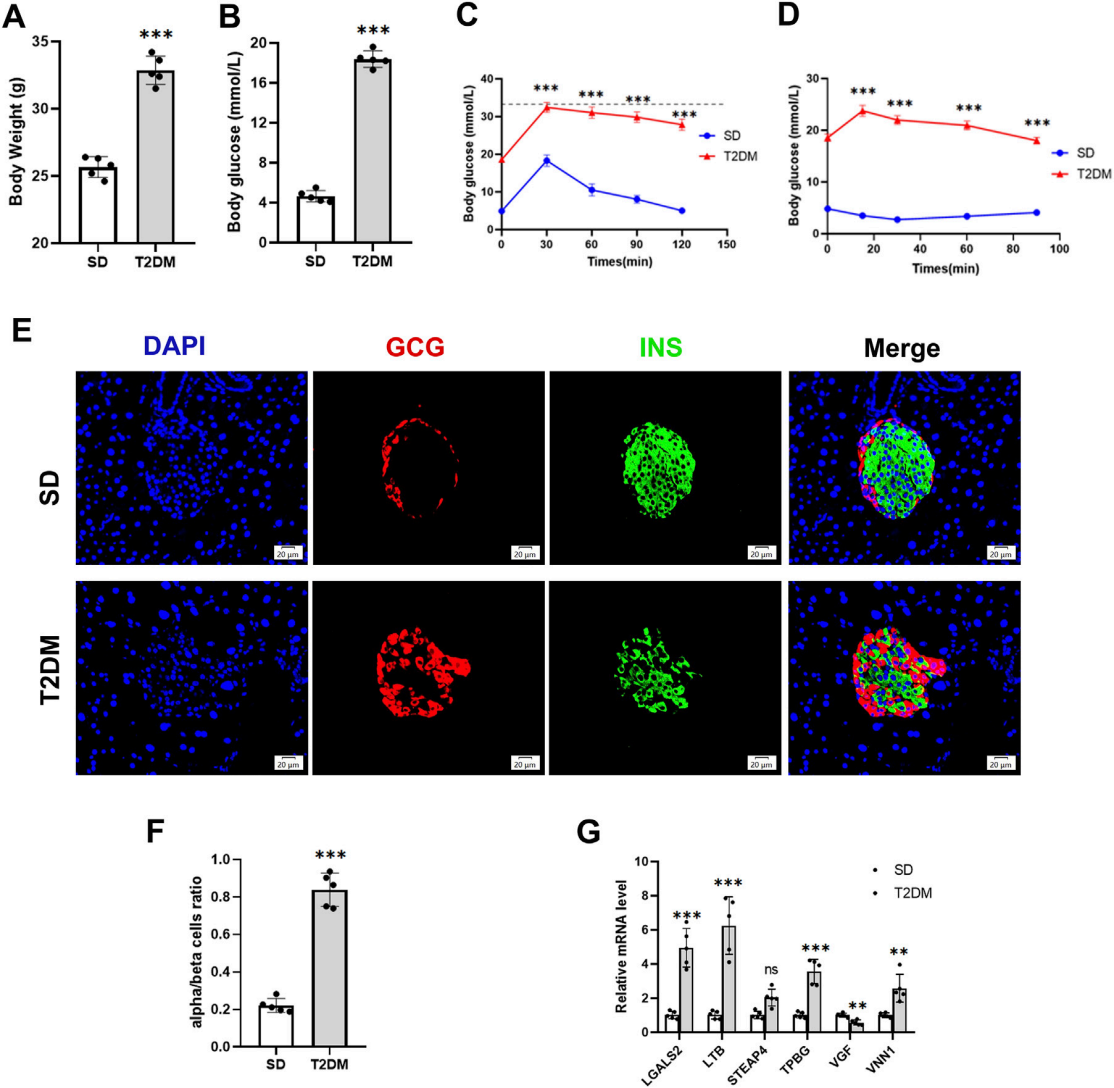
Validation of hub genes in the T2DM model. **(A)** Body weight. **(B)** Fasting blood glucose levels. **(C)** IPGTT. **(D)** IPITT. **(E and F)** Representative double-stained images of insulin and glucagon in pancreatic islets and assessment of the pancreatic α/β cell ratio. **(G)** RT-PCR analysis of hub gene expression in the pancreas of SD and T2DM mice. Scale bars = 20 μm. Values are expressed as means ± SD (n = 5). **p < 0.01, ***p < 0.001, ns, no statistical difference. T2DM, type 2 diabetes mellitus. IPGTT, intraperitoneal glucose tolerance test. IPITT, intraperitoneal insulin tolerance test.

We further validated the changes of hub genes in islets of type 2 diabetic mice. To induce a typical T2DM model, a low dose of STZ was consecutively injected intraperitoneally for HFD-fed mice. The T2DM group had significantly increased body weight, and fasting plasma glucose was significantly higher than that of the SD group (Figures 5A, B), which showed obvious impaired glucose tolerance and insulin resistance (Figures 5C, D). In addition, mice in the T2DM group had an increased number of pancreatic islet α-cells and a disproportionate α/β-cell ratio (Figures 5E, F). Interestingly, PCR results from the mouse pancreas showed that *LGALS2*, *LTB*, *TPBG*, and *VNN1* expression was upregulated, and *VGF* expression was downregulated in the T2DM group of mice compared with the SD group (Figure 5G).

**FIGURE 6:**
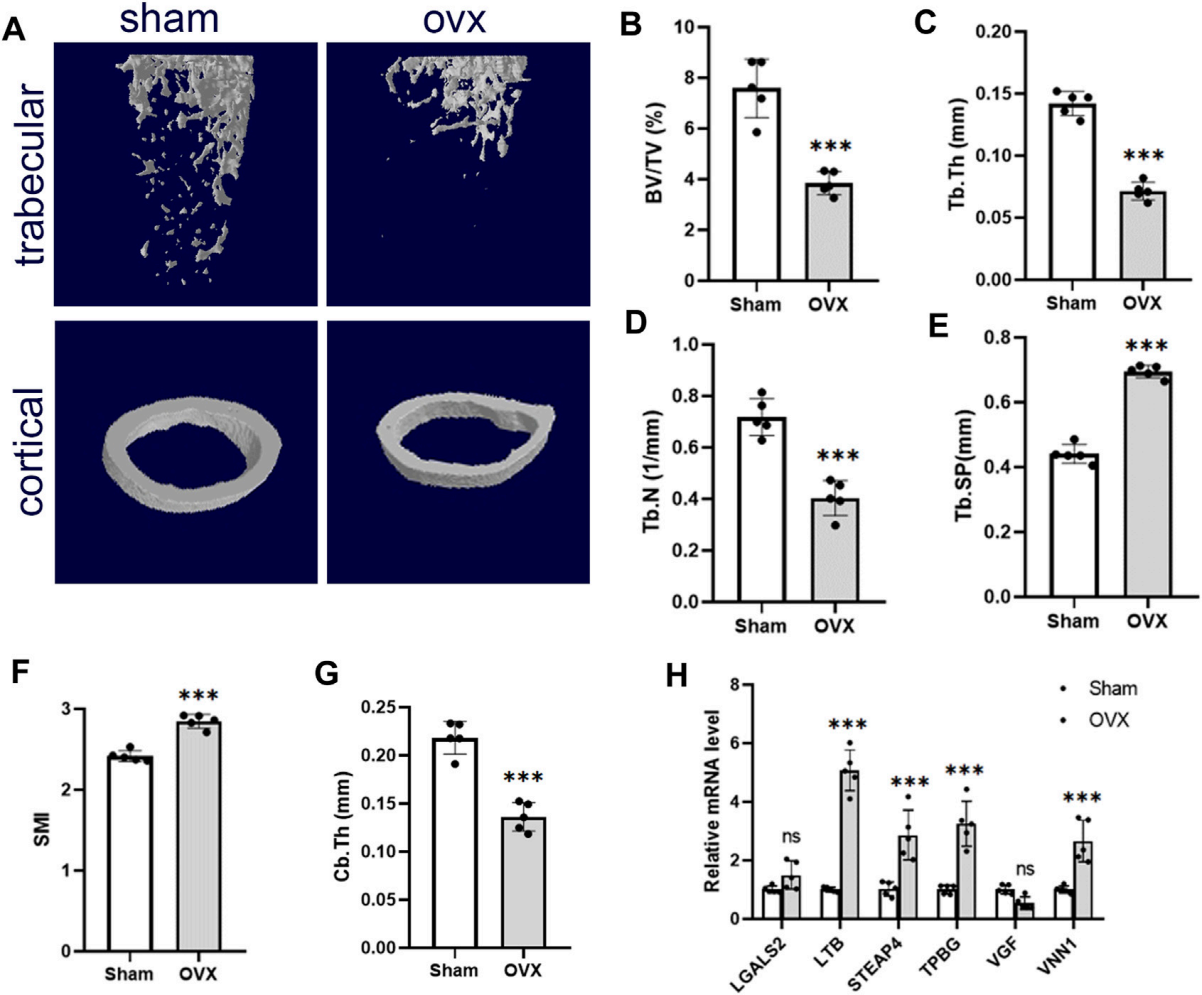
Validation of hub genes in the OP model. **(A)** Representative microcomputed tomography (μCT) images of the femur in sham and OVX mice. **(B–G)** Quantification of BV/TV, Tb.Th, Tb.N, Tb. Sp, SMI, and Cb.Th. **(H)** RT-PCR analysis of hub gene expression in bone of sham and OVX mice. Values are expressed as means ± SD (n = 5). ***p < 0.001, ns, no statistical difference. OP, osteoporosis; BV/TB, trabecular bone volume per total volume; Tb.N, trabecular number; Tb.Sp, trabecular separation; Tb.Th, trabecular thickness; SMI, structural model index; OVX, ovariectomy.

We used the OVX mice model to simulate osteoporosis in postmenopausal women. Micro-CT was used to analyze the trabecular bone changes in the distal femur of different model groups 10 weeks after OVX. Compared with the sham group, the OVX  group demonstrated a significant decrease in BV/TV, Tb.Th, Tb.N, and Cb.Th and an increase in Tb. Sp and SMI (Figures 6B–G). PCR results derived from mouse femurs showed that *LTB*, *STEAP4*, *TPBG,* and *VNN1* expression was upregulated in the OVX group of mice compared with the sham group (Figure 6H).

### 3.6 *VNN1* was upregulated in mouse models of T2DM and OP

**FIGURE 7:**
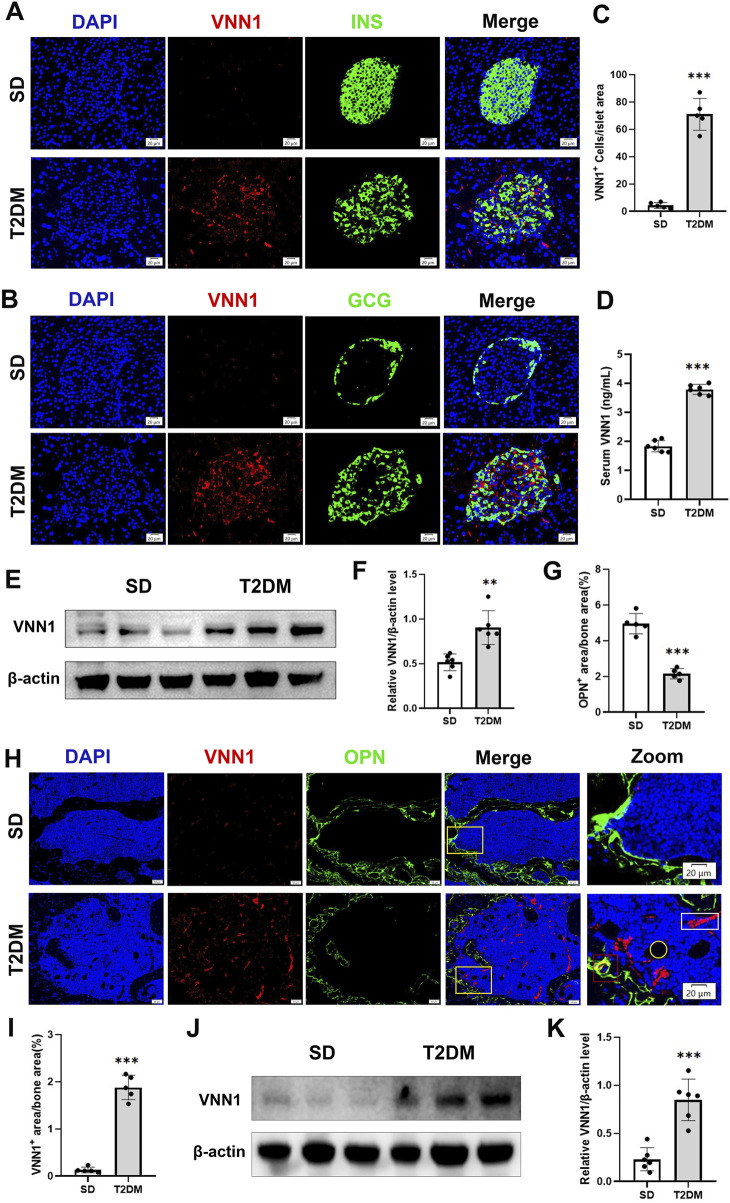
*VNN1* was upregulated in the pancreas and bone of the T2DM mouse model. Representative immunofluorescence images of insulin, glucagon, and VNN1 in pancreatic islets **(A and B)** and the number of VNN1-positive cells in pancreatic islets **(C)**. Scale bar = 20 μm. Comparison of serum VNN1 expression in mice **(D)**. Representative immunofluorescence images of OPN and VNN1 in bone **(H)** and quantitation of OPN and VNN1-positive area in the immunofluorescence assay **(G and I)**. Scale bar = 50 μm, 20 μm. Western blot showing VNN1 expression in pancreas and bone of SD and T2DM mice **(E and J)**. Quantitative analysis **(F and K)**. Values are expressed as means ± SD (n = 5 or 6). **p < 0.01, ***p < 0.001.

Through bioinformatics methods, we found that *VNN1* was an upregulated DEG in islet samples of T2DM and OP. *VNN1* is an emerging biomarker that has not yet been detected in diabetic osteoporosis patients, so we chose *VNN1* for further study. Immunofluorescence results indicated that VNN1 expression was upregulated in the T2DM group (Figures 7A–C). Western blot showed the same results, revealing a substantial increase in VNN1 protein expression in T2DM groups compared to the SD group (Figures 7E, F). To clarify the distribution location of VNN1 in the mouse pancreas, we co-stained VNN1 with insulin (INS) and glucagon (GCG). We found that VNN1 was mainly distributed in pancreatic islets and partially merged with both INS and GCG. We also examined the expression of VNN1 in the bones of mice and found that the expression of VNN1 was significantly increased in the bones of T2DM model mice (Figures 7G–K). We used the osteogenic marker osteopontin (OPN) for immunofluorescence double staining with VNN1. Zooming in on the images, we found that VNN1 was localized around osteoblasts (red rectangles), bone marrow adipocytes (yellow circles), and blood vessels (white rectangles) in the bones of T2DM model mice (Figure 7H). Similarly, serum VNN1 expression was increased in T2DM mice compared to controls (Figure 7D).

**FIGURE 8:**
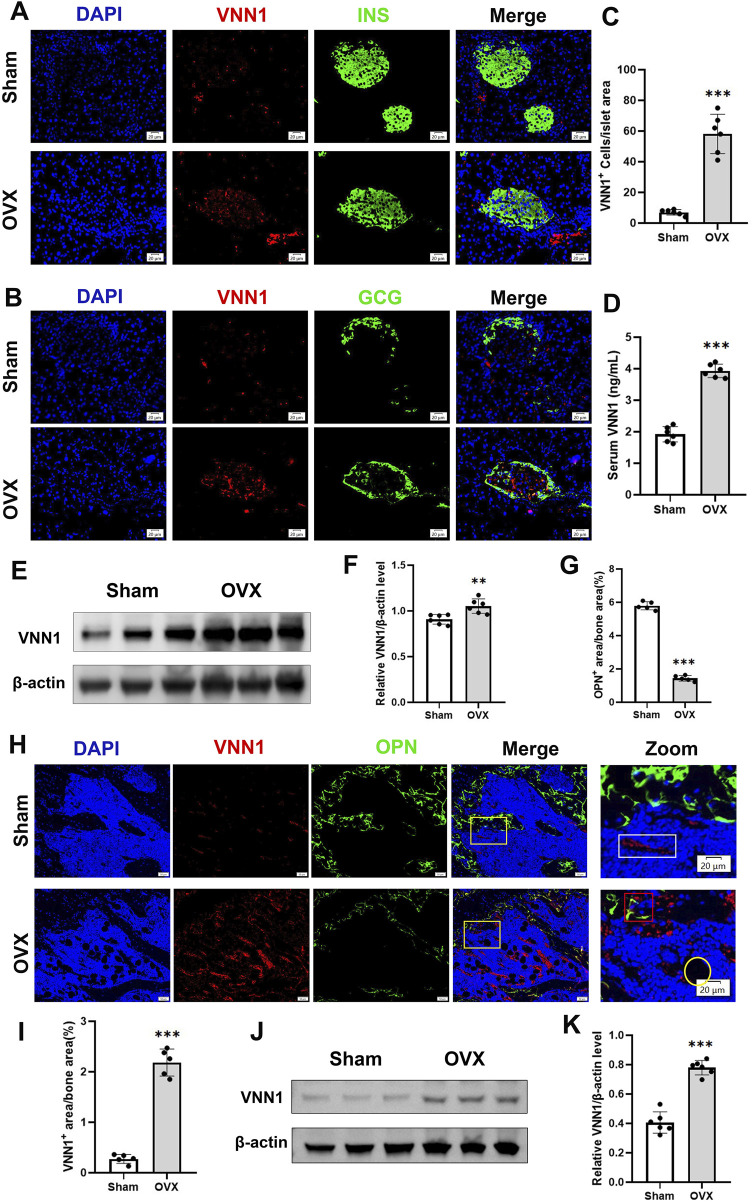
VNN1 was upregulated in the pancreas and bone of a mouse model of osteoporosis. Representative immunofluorescence images of insulin, glucagon, and VNN1 in pancreatic islets **(A and B)** and the number of VNN1-positive cells in pancreatic islets **(C)**. Scale bar = 20 μm. Comparison of serum VNN1 expression in mice **(D)**. Representative immunofluorescence images of OPN and VNN1 in bone **(H)** and quantitation of OPN and VNN1-positive area in the immunofluorescence assay **(G and I)**. Scale bar = 50 μm, 20 μm. Western blot showing the expression of VNN1 in sham and OVX mice **(E and J)**. Quantitative analysis **(F and K)**. Values are expressed as means ± SD (n = 5 or 6). **p < 0.01, ***p < 0.001.

Next, we investigated the expression of VNN1 in the pancreas and bone of OVX mice. Similarly, pancreatic VNN1 expression was increased in OVX mice (Figures 7E, F), localized predominantly in the pancreatic islets, and partially merged with INS and GCG (Figures 7A–C). VNN1 expression was increased in the serum of OVX mice compared with the sham group (Figure 8D). Immunofluorescence showed that VNN1 expression was increased in the bones of OVX mice, distributed around osteoblasts (red rectangles), bone marrow adipocytes (yellow circles), and blood vessels (white rectangles) (Figure 8H). Western blotting showed the same results, with a significant increase in VNN1 protein expression in the OVX group compared with the sham group (Figures 8J, K).

### 3.7 Verification of *VNN1* in clinical samples

**FIGURE 9:**
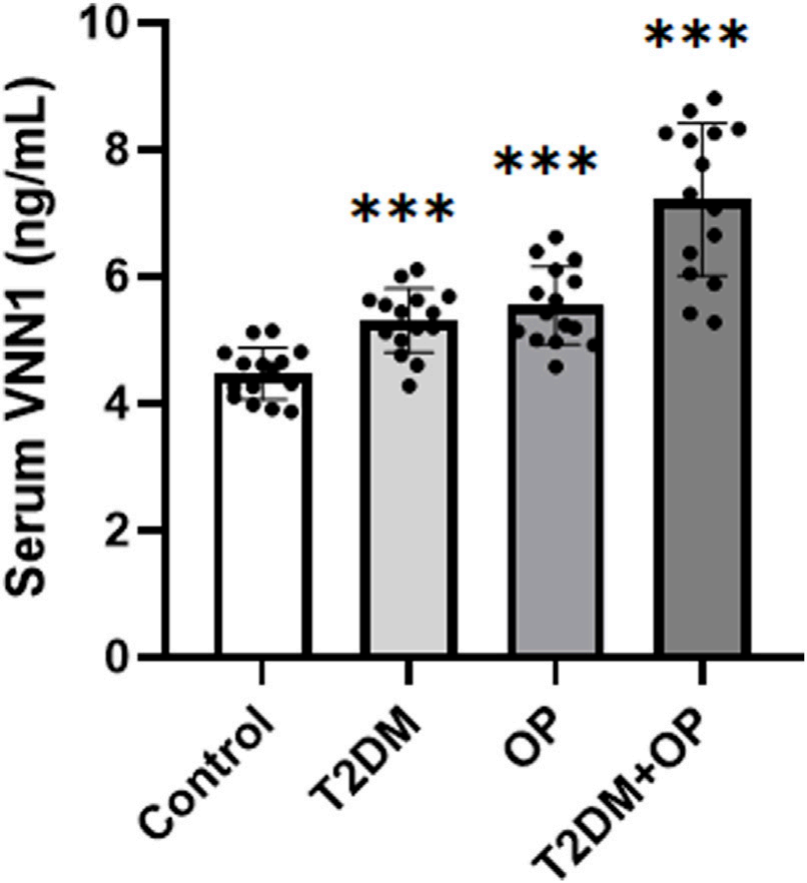
Comparison of serum *VNN1* levels among patients with T2DM, OP, T2DM and OP, and control (***p < 0.001 vs. control).

In order to verify the clinical application potential of the *VNN1* gene, the ELISA method was used to detect the protein level encoded by the *VNN1* gene in clinical samples. The T2DM group and the T2DM combined OP (T2DM + OP) group had higher blood glucose levels than the control group; the DXA femoral T-score was lower in the OP group and the T2DM + OP group than the control group, and the difference was statistically significant (Table 2). The expression of serum *VNN1* in the T2DM, OP, and T2DM + OP groups was higher than that in the control group, and the difference was also statistically significant (Figure 9). These findings indicate that *VNN1* is a potential biomarker for the early diagnosis and prognostic prediction of T2DM and OP.

**TABLE 2:** Comparison of data of volunteers in four groups.

	Control (n = 15)	T2DM (n = 15)	OP (n = 15)	T2DM + OP (n = 15)
Age (years)	46.07	52.60	54.47	57.33
FBG (mmol/L)	4.90	9.41***	4.89	10.22***
Femoral neck T-score	0.38	0.29	−2.79***	−2.88***
*VNN1* (ng/ml)	4.47	5.31***	5.54***	7.22***

(***p < 0.001 vs. control).

## 4 Discussion

In recent years, the relationship between T2DM and osteoporosis has received widespread attention (Napoli et al., 2017; Greenhill, 2018). In this study, we obtained the T2DM and OP datasets from GEO, identified the common differentially expressed genes (co-DEGs) of T2DM and OP, and constructed the coexpression network based on weighted gene coexpression network analysis (WGCNA). GO analysis of these 19 co-DEGs showed that these genes were mainly involved in processes such as cell–cell adhesion, positive regulation of I-κB kinase/NF-κB signaling, and signal transduction, and were enriched in pantothenate and CoA biosynthesis and the glycosaminoglycan biosynthesis-chondroitin sulfate/dermatan sulfate and the renin–angiotensin systems. Then, based on the LASSO algorithms, we identified six hub genes, namely, *LGALS2*, *LTB*, *STEAP4*, *TPBG*, *VGF*, and *VNN1*. Next, we verified the expression of these central genes in T2DM and OP mouse models and patients using RT-PCR, immunofluorescence, Western blot, and ELISA. We finally identified *VNN1* as the most diagnostic hub gene.

Vanin-1 (*VNN1*) is a pantethionase enzyme that is anchored to the extracellular membrane of epithelial and myeloid cells, hydrolyses pantethionine, and produces vitamin B5 and cysteamine (Pitari et al., 2000; Kang et al., 2016). *VNN1* has been reported to exacerbate paraneoplastic islet dysfunction by regulating environmental stress, viability, and functionality of islet cells and increasing oxidative stress and cysteamine, leading to the deterioration of the microenvironment of insulin-secreting cells (Kang et al., 2016). Animal experiments demonstrated that hepatic VNN1 expression was induced in db/db and diet-induced obese mice exhibiting severe hepatic steatosis and that liver-specific knockdown of *VNN1* ameliorated hepatic steatosis in these animal models (Chen et al., 2014). Clinical investigations indicated that the levels of VNN1 were increased in the urine and blood of diabetic patients (Fugmann et al., 2011). Consistent with the clinical findings, our study shows that VNN1 is elevated in the serum of T2DM mice and humans. Surprisingly, our study found that VNN1 expression was increased in the pancreas of OVX mice, which may be associated with insulin resistance due to postmenopause.

In addition, *Vanin-1* knockout corrected the increase in chondrogenesis in ank/ank (an animal model of spondyloarthropathy) bone marrow mesenchymal stromal cells (BMSCs) as well as the increase in chondrogenic transdifferentiation and calcification in ank/ank aortic smooth muscle cell explants (Johnson et al., 2008). This finding suggests the potential research value of VNN1 in bone disease. However, VNN1 has been less studied in osteoporosis, and our study examined VNN1 levels in patients and mice with osteoporosis. VNN1 expression was significantly increased in bone and serum from osteoporotic and diabetic mice, as well as in serum from patients with osteoporosis and diabetes. Notably, serum levels of VNN1 were higher in patients with both T2DM and OP than in patients with only one of these diseases. Therefore, VNN1 can be used as a marker for the detection of diabetic osteoporosis, which is the clinical significance of this study.

In the enrichment analysis section, we noted that the “renin-angiotensin system” is involved in the crosstalk between T2DM and OP. The renin–angiotensin system (RAS) is a fluid and electrolyte regulatory system that is essential for maintaining blood volume, blood pressure, and glucose homeostasis (Luther and Brown, 2011). Several previous studies have also shown that the classical anti-inflammatory axis of the RAS, ACE2/A1-7/Mas, exerts a protective effect on pancreatic β-cell function under metabolic stress by improving microcirculation, modulating mitochondrial function, and inhibiting oxidative stress, as well as reducing apoptosis and dedifferentiation (Xuan et al., 2018; Ma et al., 2020; Chen et al., 2023a). Furthermore, it has been demonstrated that Ang (1–7) improves abnormal bone metabolism and micro-architecture by increasing mineralization while inhibiting osteoclastogenesis (Zhang et al., 2016; Queiroz-Junior et al., 2019). The ACE2/Ang1-7/Mas receptor axis counteracts the effects of pro-inflammatory cytokines (IL-6 and TNF-α) to improve bone health (Mkhize et al., 2023). Upregulation of the pro-inflammatory component of RAS is positively correlated with T2DM and may also alter the bone microenvironment by altering the bone marrow inflammatory status and reducing BMD by shifting the osteoprotegerin (OPG)/nuclear factor kappa-Β ligand (RANKL) ratio (Mkhize et al., 2023). Thus, an imbalance in the renin–angiotensin system may play a key role in the pathogenesis of T2DM and OP.

## Data Availability

The raw data supporting the conclusion of this article will be made available by the authors, without undue reservation.
