# Supplementary figures and images for: Integrated analysis of genes shared between type 2 diabetes mellitus and osteoporosis
Source: Front Pharmacol. 2024 Jun 20;15:1388205. doi: 10.3389/fphar.2024.1388205 (PMC11222565; doi:10.3389/fphar.2024.1388205)

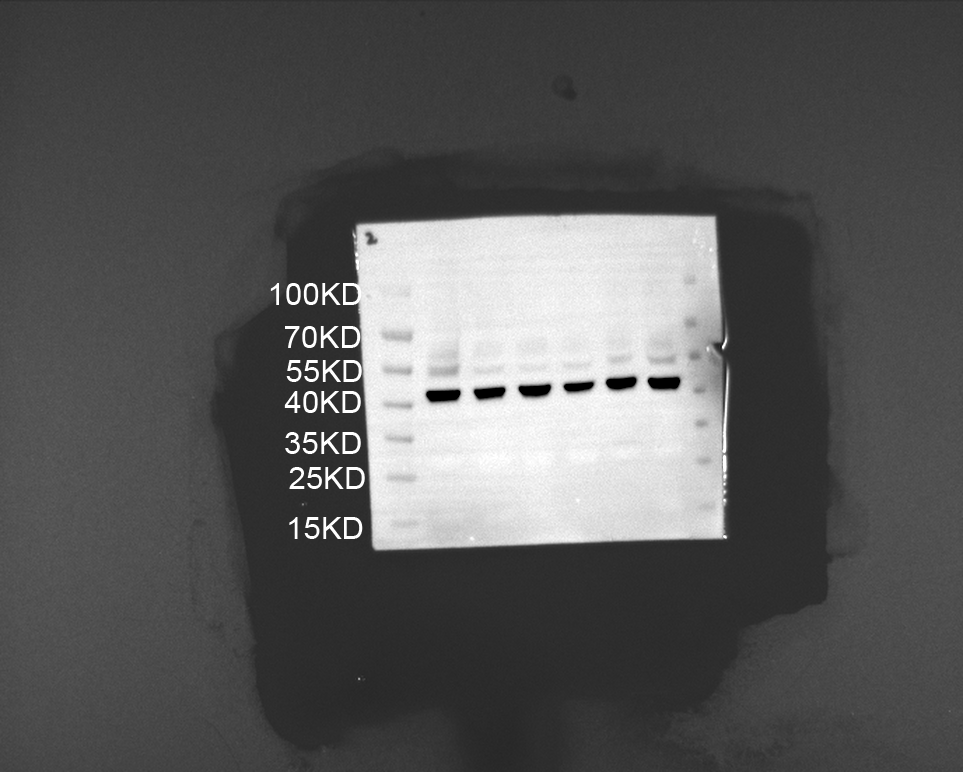

Supplement: Supplementary file 2 [file DataSheet1.ZIP › Data/boneactin.tif]

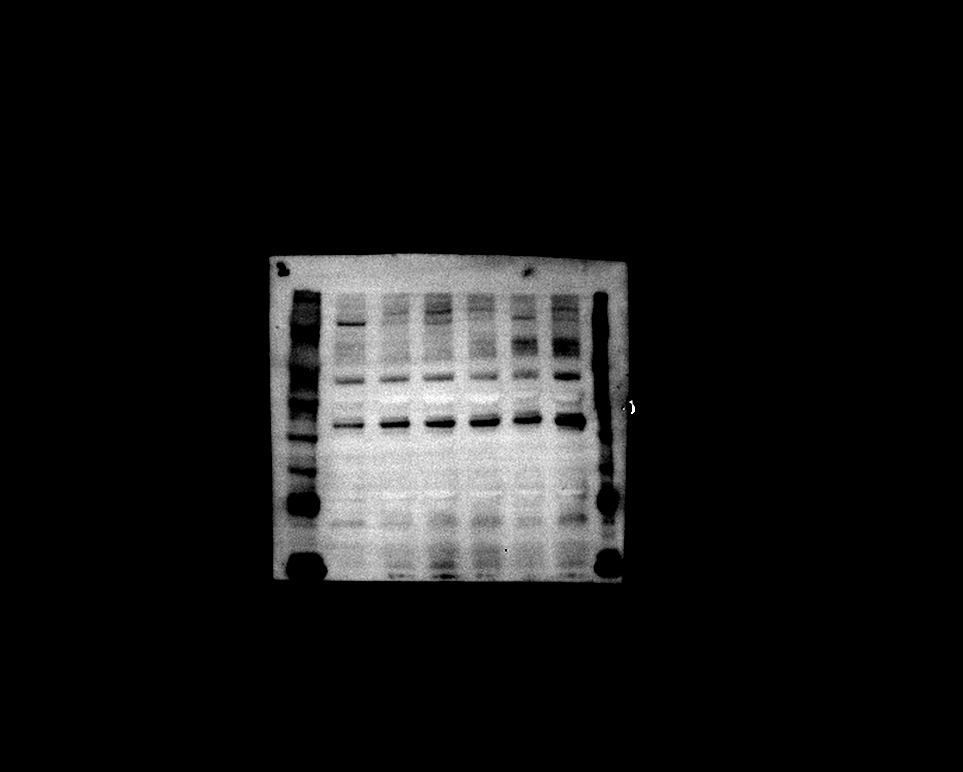

Supplement: Supplementary file 2 [file DataSheet1.ZIP › Data/bonevnn1.tif]

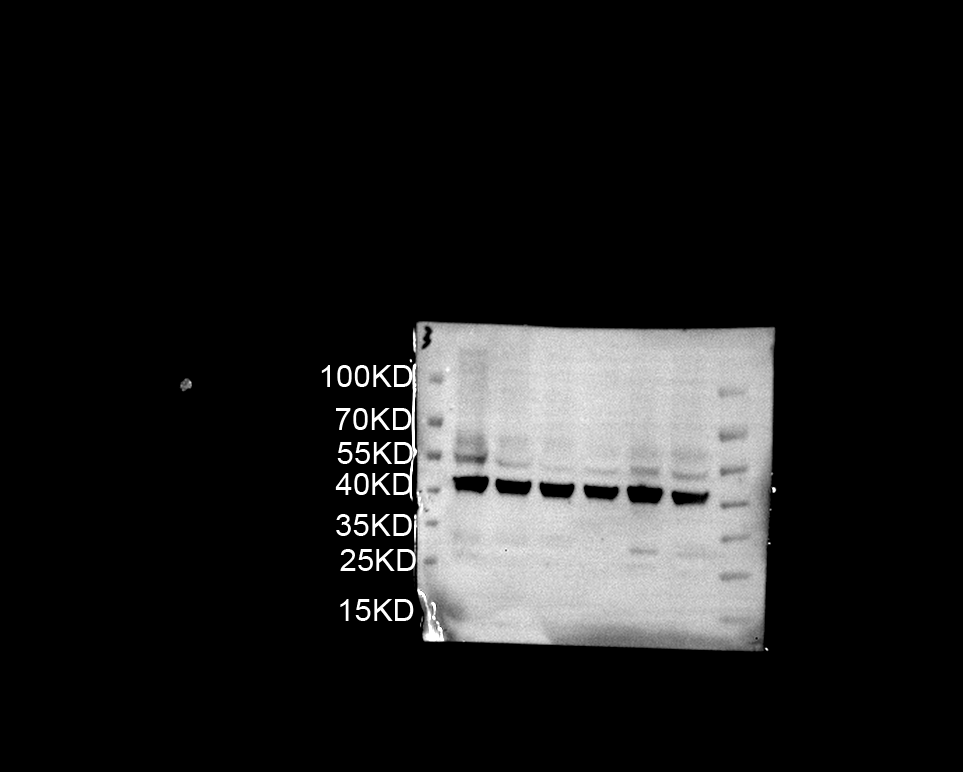

Supplement: Supplementary file 2 [file DataSheet1.ZIP › Data/isletactin.tif]

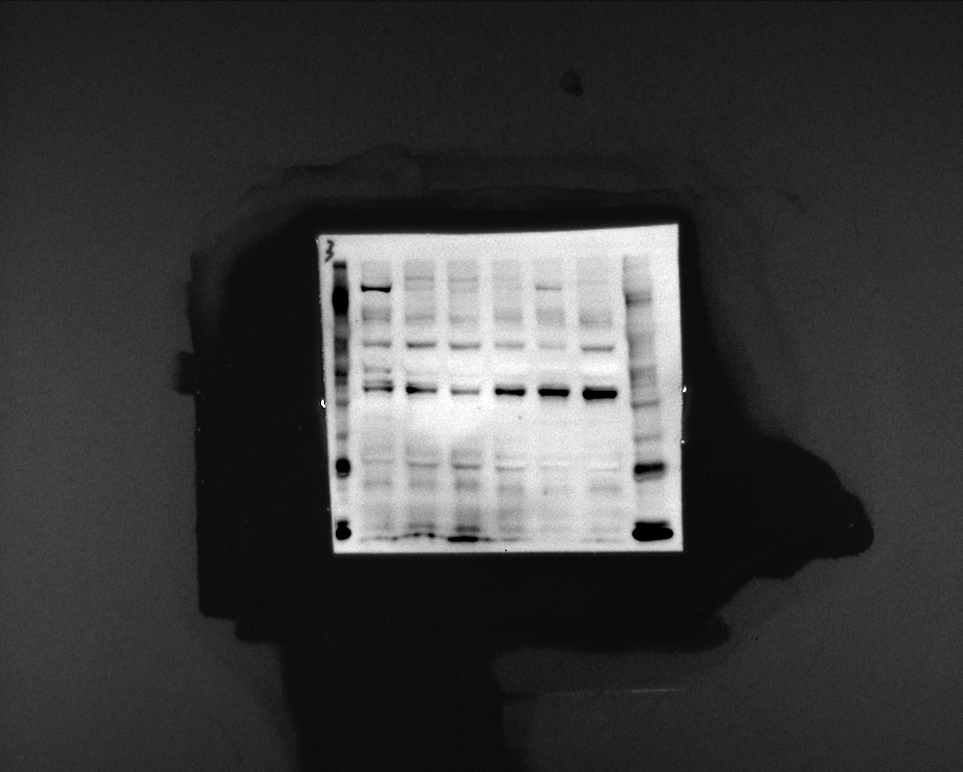

Supplement: Supplementary file 2 [file DataSheet1.ZIP › Data/isletvnn1.tif]

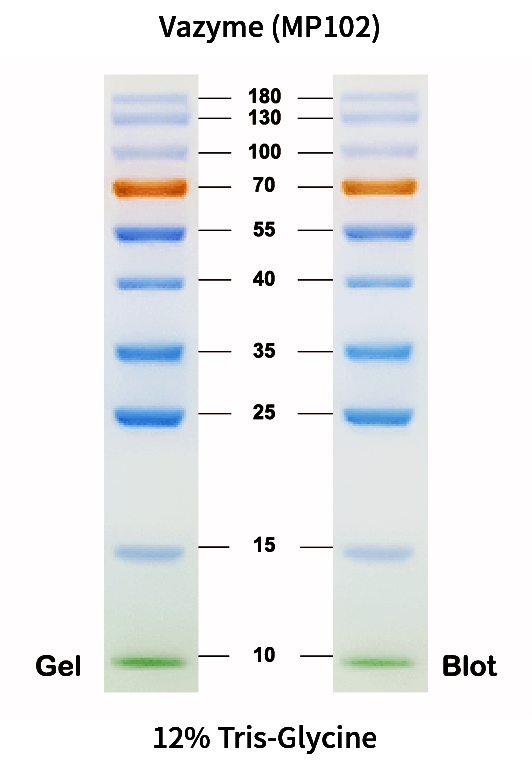

Supplement: Supplementary file 2 [file DataSheet1.ZIP › Data/Ladder.png]

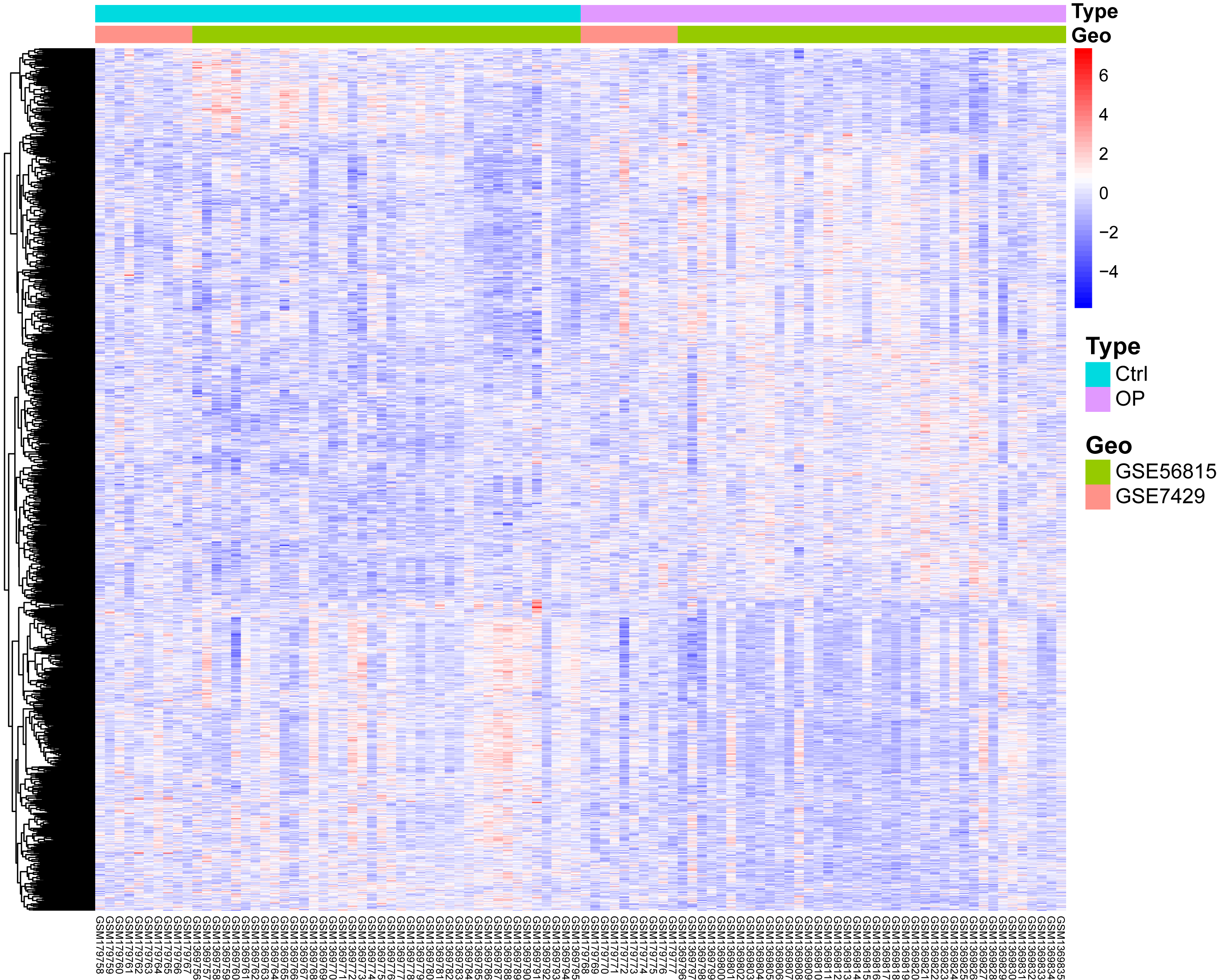

Supplement: Supplementary file 3 [file Image2.TIF]

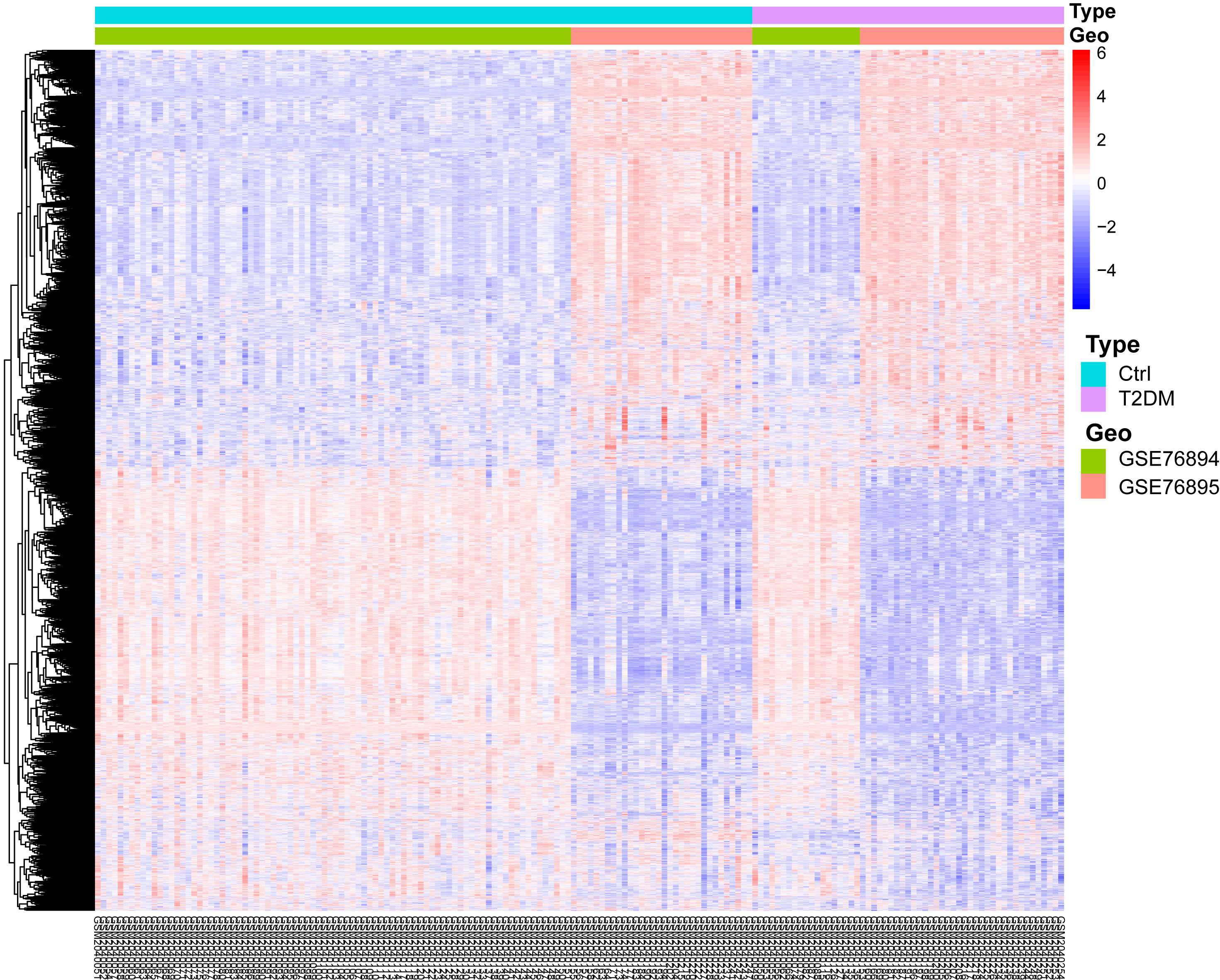

Supplement: Supplementary file 4 [file Image1.TIF]

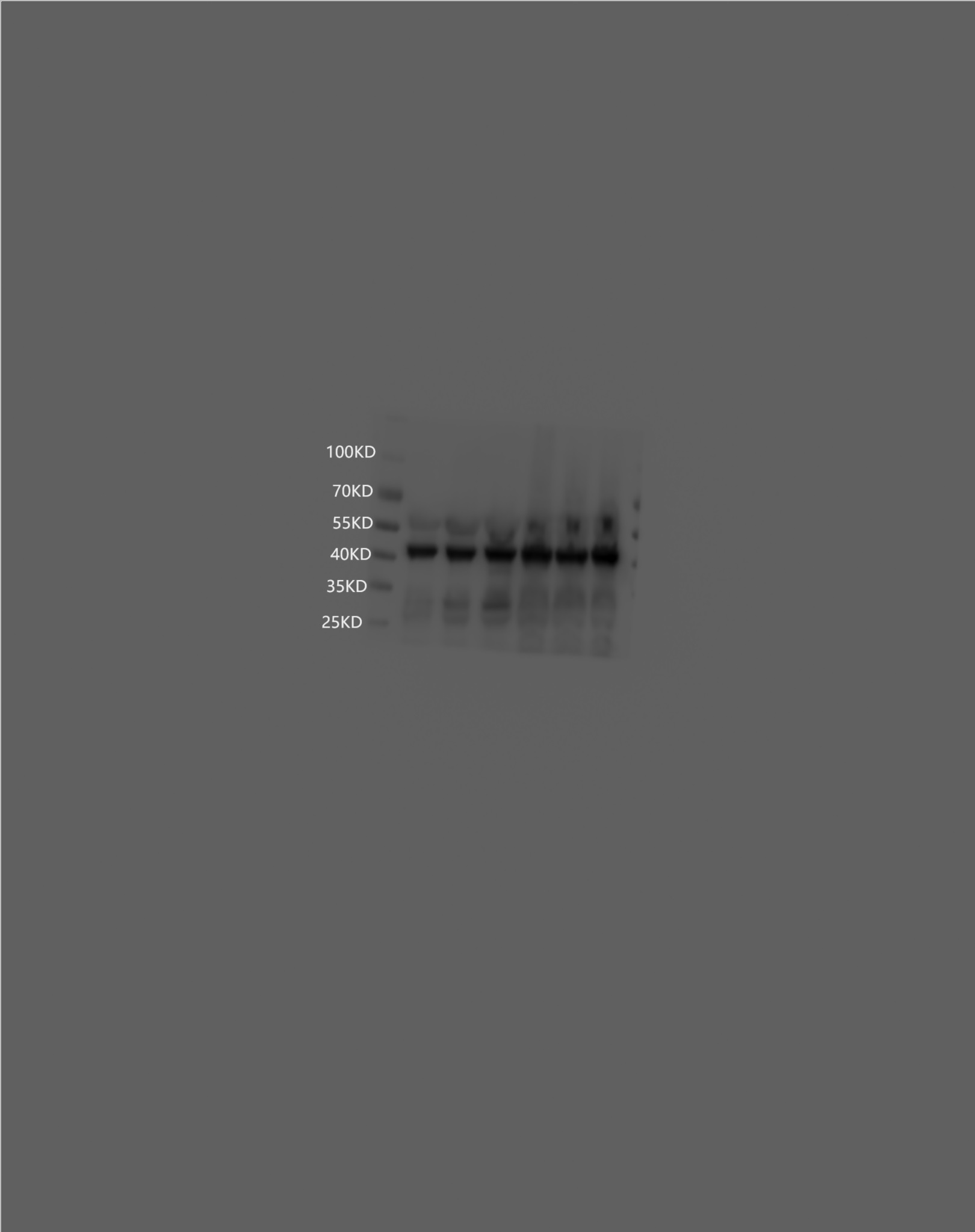

Supplement: Supplementary file 5 [file DataSheet2.ZIP › Data/OVXisletactin.jpg]

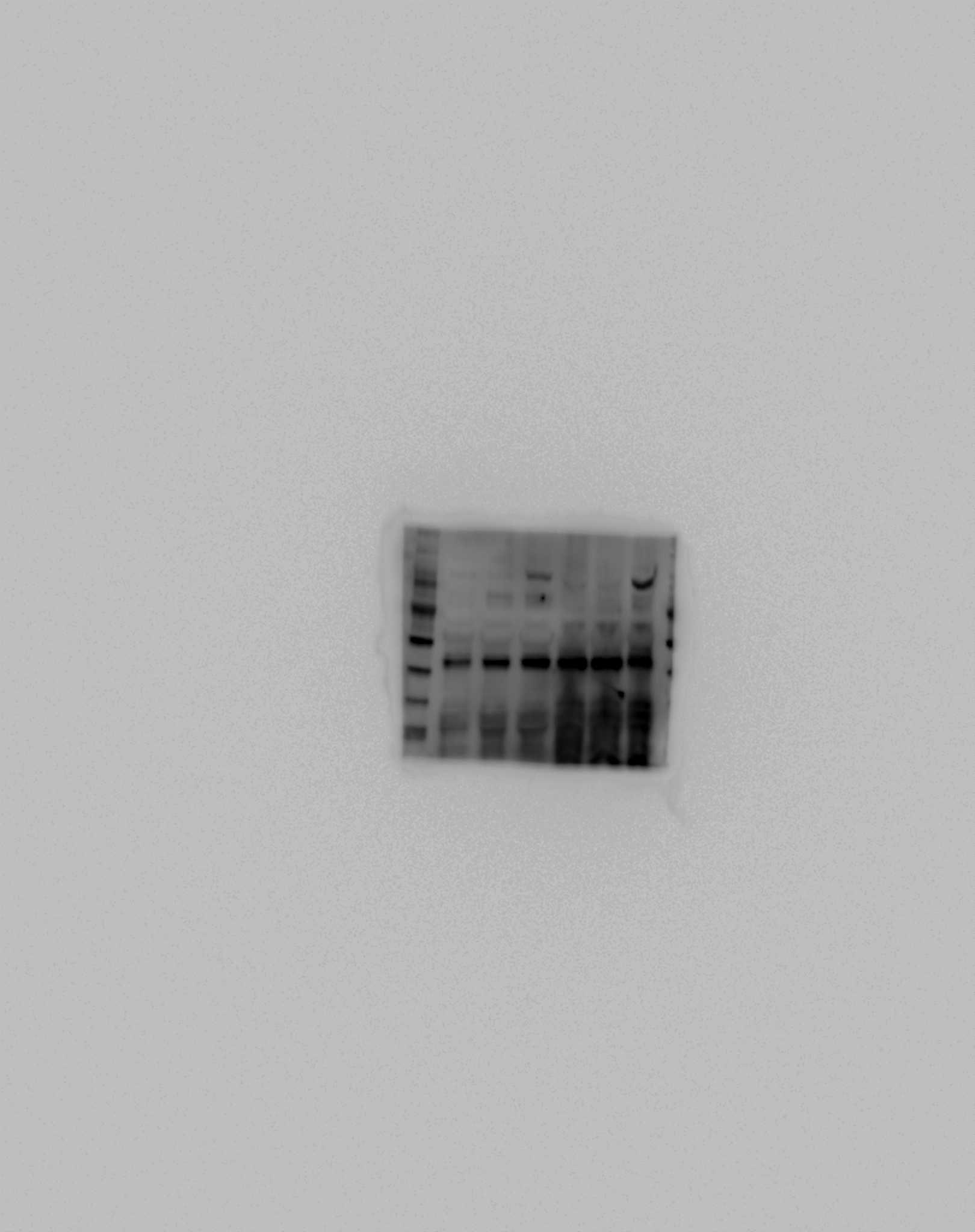

Supplement: Supplementary file 5 [file DataSheet2.ZIP › Data/OVXisletVNN1.jpg]

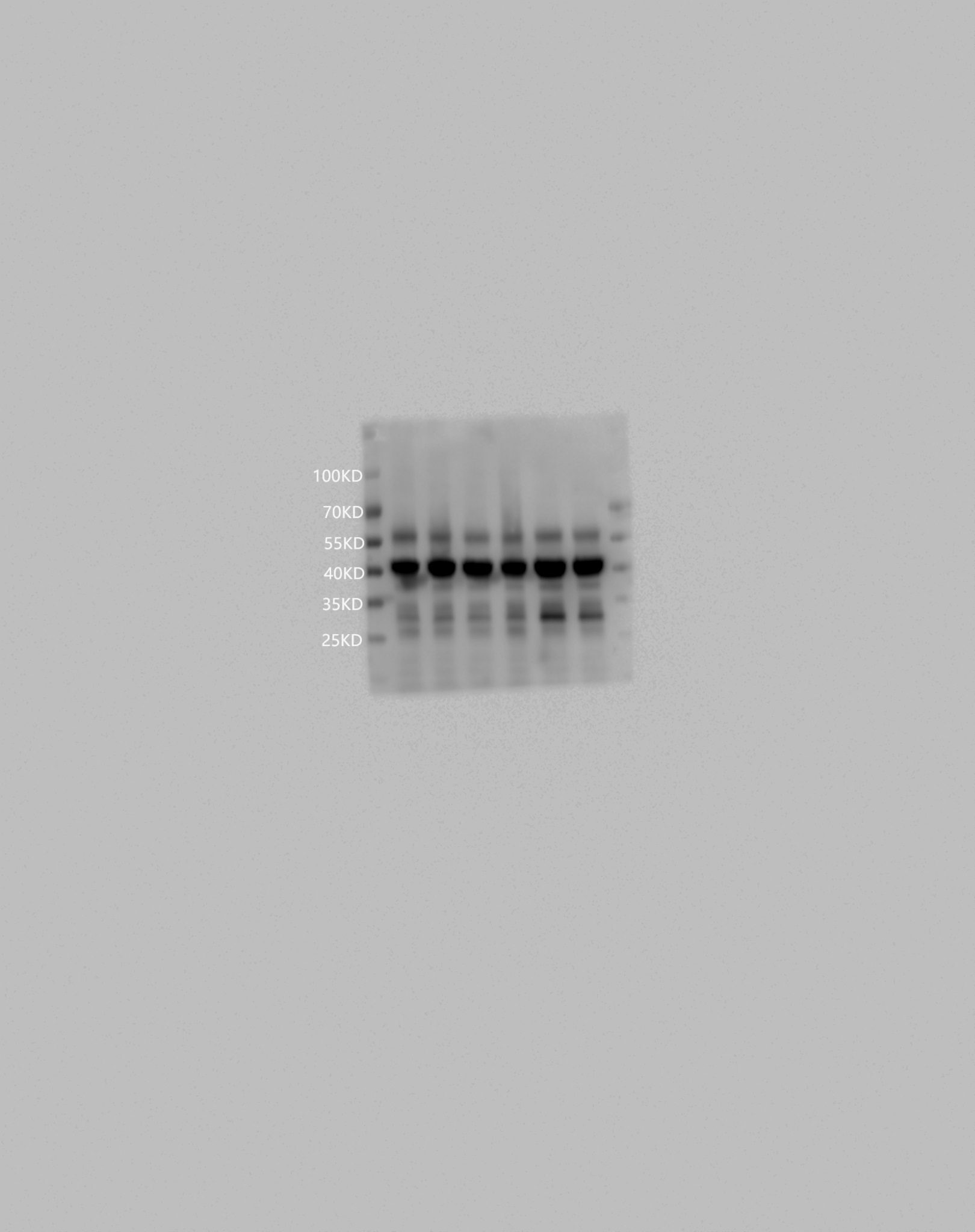

Supplement: Supplementary file 5 [file DataSheet2.ZIP › Data/T2DMboneactin.jpg]

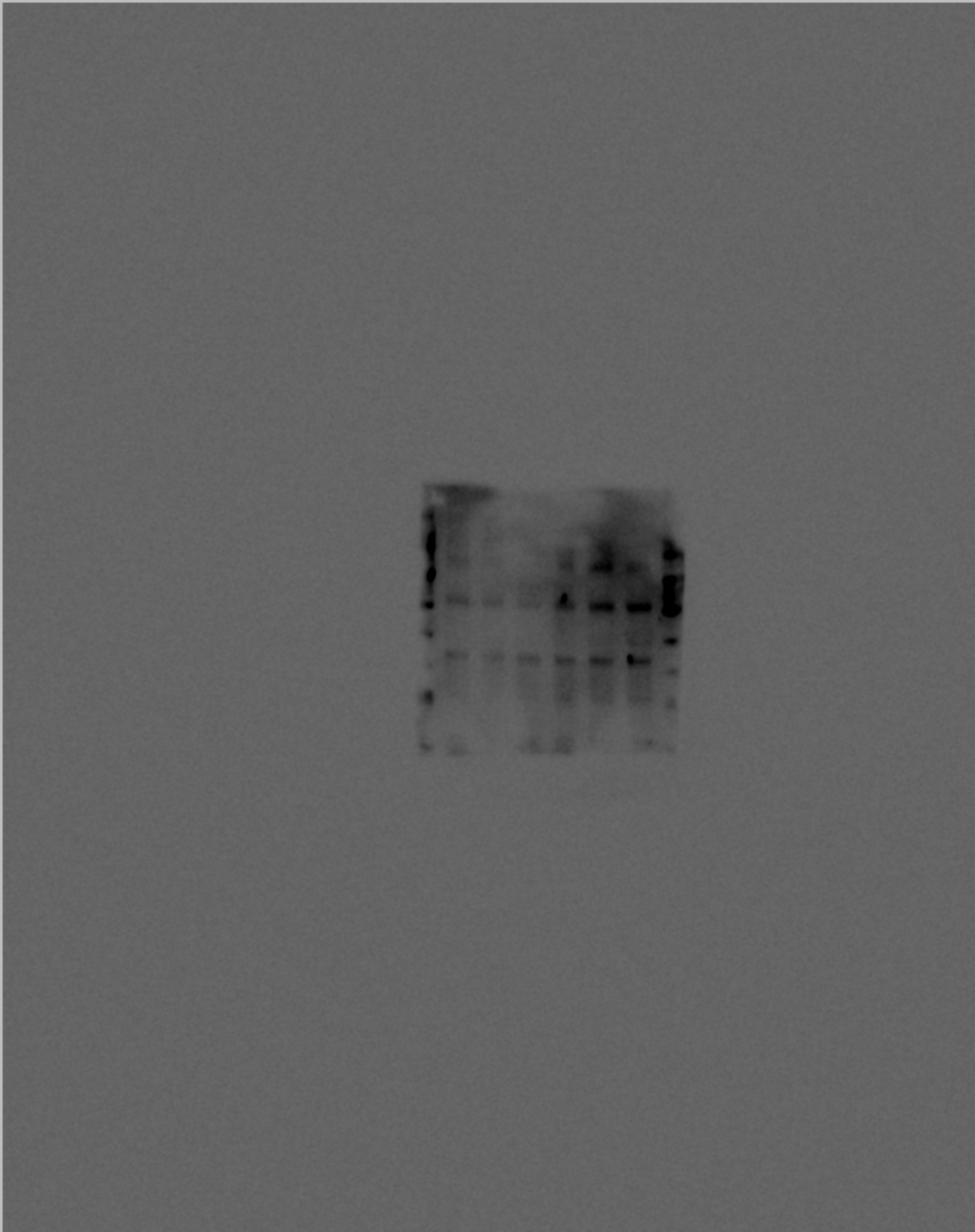

Supplement: Supplementary file 5 [file DataSheet2.ZIP › Data/T2DMboneVNN1.jpg]
